# Screening of Twelve Pea (*Pisum sativum* L.) Cultivars and Their Isolates Focusing on the Protein Characterization, Functionality, and Sensory Profiles

**DOI:** 10.3390/foods10040758

**Published:** 2021-04-02

**Authors:** Verónica García Arteaga, Sonja Kraus, Michael Schott, Isabel Muranyi, Ute Schweiggert-Weisz, Peter Eisner

**Affiliations:** 1 Fraunhofer Institute for Process Engineering and Packaging IVV, 85354 Freising, Germany; sonni.kraus@gmail.com (S.K.); michael.schott@ivv.fraunhofer.de (M.S.); isabel.muranyi@ivv.fraunhofer.de (I.M.); uweisz@uni-bonn.de (U.S.-W.); peter.eisner@ivv.fraunhofer.de (P.E.); 2 Center of Life and Food Sciences Weihenstephan, Technical University of Munich, 85354 Freising, Germany; 3 Institute for Nutritional and Food Sciences, University of Bonn, 53012 Bonn, Germany; 4 ZIEL—Institute for Food & Health, Technical University of Munich, 85354 Freising, Germany; 5 School of Technology and Engineering, Steinbeis-Hochschule, 12489 Berlin, Germany

**Keywords:** pea (*Pisum sativum* L.), spray-dry, functional properties, sensory profile, protein characterization, pea allergens

## Abstract

Pea protein concentrates and isolates are important raw materials for the production of plant-based food products. To select suitable peas (*Pisum sativum* L.) for protein extraction for further use as food ingredients, twelve different cultivars were subjected to isoelectric precipitation and spray drying. Both the dehulled pea flours and protein isolates were characterized regarding their chemical composition and the isolates were analyzed for their functional properties, sensory profiles, and molecular weight distributions. Orchestra, Florida, Dolores, and RLPY cultivars showed the highest protein yields. The electrophoretic profiles were similar, indicating the presence of all main pea allergens in all isolates. The colors of the isolates were significantly different regarding lightness (L*) and red-green (a*) components. The largest particle size was shown by the isolate from Florida cultivar, whereas the lowest was from the RLPY isolate. At pH 7, protein solubility ranged from 40% to 62% and the emulsifying capacity ranged from 600 to 835 mL g^−1^. The principal component analysis revealed similarities among certain pea cultivars regarding their physicochemical and functional properties. The sensory profile of the individual isolates was rather similar, with an exception of the *pea-like* and *bitter* attributes, which were significantly different among the isolates.

## 1. Introduction

Peas (*Pisum sativum* L.) were domesticated around 10,000 years ago. Over the years, evolution and breeding has influenced the number of pea cultivars found today. In Europe, according to the Food and Agriculture Organization database [1], France and Germany were the biggest dry pea seed producers in 2019. The differences among cultivars depend on their cultivated status (wild or cultivated), geographical origin, and usage (fresh or dry) [2]. The study of different cultivars, their breeding, and their inclusion in the genome database is a continuous process [3]. From an agronomic point of view, cultivation factors such as maximum yield security, plant stability, seed percentage, and protein yield are the most important characteristics considered for pea cultivation; however, for industrial food production, factors such as protein content, functionality, taste, and color are also considered [4]. Peas contain high amounts of protein at around 20–35%, low amounts of fat at around 0.5–4.0%, and high amounts of starch at around 30–48% [5,6,7]. Previous studies have investigated the differences in pea cultivar compositions and have found environmental and genotypic variations as the main factors for the described data discrepancies. The aroma of the pea seeds also changes significantly depending on the cultivar, harvest year, and processing conditions [8,9].

Vegetarian or vegan diets might lead to protein deficiencies, making peas an interesting protein source for plant-based food products [10]. According to the Global Market Insights report [11], the pea protein market is estimated to grow by 12% compound annual growth rate (CAGR) by 2026. The main proteins in peas correspond to storage proteins. These are divided into globulins and albumins, corresponding to 55–80% and 18–25%, respectively, depending on genetic and environmental factors [6,12,13]. Similar to other legumes, the major globulins in peas are divided into 7S vicilin–convicilin and 11S legumin fractions [14]. The molecular structures and weight distributions are different among these proteins. Legumin is a hexamer with major polypeptide subunits of ~40 and ~20 kDa, which can be bound by disulfide bonds. Vicilin is a trimer (each subunit ~50 kDa) lacking cysteine residues that can undergo post-translational proteolysis, resulting in different fractions. Convicilin is a trimer (~70 kDa) without any translational modification [15,16].

Pea proteins are used as concentrates (40–90% protein) and isolates (>90% protein) in the food industry; however, the extraction of pea protein isolate (PPIs) at laboratory and pilot scales has shown protein contents of around 80–90% [17,18,19]. These studies have found that depending on the cultivar and the extraction method, the protein solubility and emulsifying and foaming capacity were significantly affected; however, Stone and Karalash [17] concluded that overall, the extraction method has a greater influence than the cultivar. The PPIs investigated in the above-mentioned studies showed higher functionality than commercial isolates. This could be due to a deviating production or drying process. Industrial protein ingredient suppliers usually use spray drying, whereas lyophilization is mainly used for scientific purposes at the laboratory scale. Spray drying might affect the aroma and protein structure, and thus the protein profile, particle size, and functionality [9,20]. Moreover, most authors have investigated cultivars available in their countries. In Germany, the cultivars Astronaute and Salamanca are mainly used because of their high seed yields [21]; however, to our knowledge, only protein preparations of the latter cultivar have been characterized scientifically [22]. A broader screening of European pea cultivars would increase the ability to select a cultivar that fulfills specific product needs.

Another reason for the high popularity of peas as raw materials for protein isolation is that unlike soy, pea proteins do not need to be declared as allergens in Europe. However, two major allergens, namely convicilin (Pis s 2) and vicilin (Pis s 1), have been identified [23]. Pis s 2 corresponds to a 62–67 kDa fraction, whereas Pis s 1 corresponds to 47–50 kDa (mature vicilin-αβγ) and 32 kDa (vicilin-αβ) fractions. These allergens could potentially promote cross-reactions with other legume allergens; thus, recent studies suggest their inclusion in the allergen declaration list [24,25]. The allergenic potential might vary within and among cultivars, as they have shown significant proteomic variations of the same pea cultivar harvested over three consecutive years [16].

The present study aimed to investigate pea cultivars grown in Germany and France, regarding chemical compositions of their flours and isolates, as well as the protein yields, functional properties, aroma profiles, and molecular weight distribution of the PPIs. Among the data assessed, this study aimed to identify PPIs of cultivars showing similar chemical, functional, and sensory properties in order to use them in combination or interchangeably in the food industry, without having significant effects on the final product quality.

## 2. Materials and Methods

### 2.1. Materials

The different field pea seeds (*Pisum sativum* L.) were kindly provided by Norddeutsche Pflanzenzucht Hans-Georg-Lembke KG (Holtsee, Germany) and are shown in Table 1. The Broad Range™ Unstained Standard, 4–20% Criterion™ TGX Stain-Free™ Precast Gels, and Coomassie blue R-250 were purchased from Bio-Rad Laboratories GmbH (Feldkirchen, Germany). Sodium dihydrogen phosphate, sodium dodecyl sulfate, and sodium monohydrogen phosphate were purchased from Sigma-Aldrich Chemie GmbH (Munich, Germany). All chemicals used in this study were of analytical grade.

### 2.2. Production of Pea Flour

Peas were dehulled and split using an underflow peeler (Streckel and Schrader KG, Hamburg, Germany). The kernels were separated using a zig-zag airlift system and milled with a pilot plant impact mill with 0.5 mm sieve insertion (Alpine Hoakawa AG, Augsburg, Germany).

### 2.3. Production of Pea Protein Isolate

The isolation of pea protein was performed according to Tian and Kyle [26] following an alkaline extraction with isoelectric precipitation (AE-IEP) with some changes. An aqueous alkaline extract of the pea flour was prepared in deionized (DI) water at a ratio of 1:5 (*w*/*w*) at pH 8.0 using 3.0 mol/L NaOH, which was stirred for 60 min. The protein extract was sieved (0.8 mm) after centrifugation at 8000× *g* for 20 min at 15 °C (8K, Sigma Laborzentrifugen GmbH, Osterode am Harz, Germany). For isoelectric precipitation, the protein extract was adjusted to pH 4.5 using 3.0 mol/L HCl and left overnight at 4 °C. The precipitated proteins were separated by centrifugation at 8000× *g* for 20 min at 15 °C and the protein isolate was dispersed in DI water to a dry matter content of 8%. After neutralization to pH 7.0, the isolate dispersion was homogenized at 11,000 rpm for 2 min using an Ultraturrax (IKA^®^-Werke GmbH and Co. KG, Staufen, Germany) prior to spray drying. The spray drying was performed using a Mini Spray Dryer B-191 (BUCHI Labortechnik GmbH, Essen, Germany) at inlet and outlet temperatures of 180 °C and 80 °C, respectively, as well as with a 95% aspirator output. The spray-dried isolates were used for further analysis. The protein yield was calculated as grams of protein per kilogram of seeds. Due to the limited amounts of pea seeds, the protein extractions and spray drying were performed once. We assumed that the protein extraction and yield values are representative of the process, as other studies have shown low standard deviations in their own extractions [17,18].

### 2.4. Chemical Composition

The analysis of the chemical compositions of the pea flours and PPIs included determination of the dry matter, ash, protein, starch, and fat contents. 

Dry matter and ash contents were determined using thermogravimetric methods (TGA 701, Leco Instruments, Mönchengladbach, Germany). The protein content was determined according to the Dumas combustion method (TruMac N, Leco Instruments, Mönchengladbach, Germany) using the average nitrogen-to-protein conversion factor of N × 6.25. All analyses were performed in duplicate and in accordance with the Association of Official Analytical Collaboration (AOAC) Official Methods [27,28].

The starch content was determined in duplicate using a Starch UV-Test Kit according to the manufacturer’s instructions (R-Biopharm AG, Darmstadt, Germany). The fat content was determined according to the Caviezel method [29] with some modifications. In extraction vessels, 2–3 g of the sample was mixed with 1.5 g potassium hydroxide, 5 mL stock solution, and 40 mL 1-butanol. After separation of the derivatized fatty acids by gas chromatography (GC 7890A, Agilent Technologies Germany GmbH & Co. KG, Waldbronn, Germany), the total fat content was determined by summing up all detected methyl esters in relation to an internal standard. Mazola corn germ oil served as the reference. The results are given in fat%, calculated as methyl ester.

### 2.5. Molecular Weight Distribution Using Sodium Dodecyl Sulfate–Polyacrylamide Gel Electrophoresis (SDS-PAGE)

The molecular weight distribution was analyzed using SDS-PAGE under non-reducing and reducing conditions according to the method used by Laemmli [30], with slight modifications. Briefly, 5 μg/μL protein solution (based on dry matter) was prepared in 1× treatment buffer (50% (*v*/*v*) 2× Tris-HCl treatment buffer, 50% (*v*/*v*) phosphate buffer (pH 7)). The 2× treatment buffer was prepared using 0.125M from the 4× stacking gel buffer (0.5M Tris, adjusted with HCl to pH 6.8), 4% from 10% SDS, 20% glycerol, and 0.02% Bromophenol Blue, while for reduction conditions 0.2M dithiothreitol was added. The samples were heated (95 °C, 5 min) prior to centrifugation at 12,045× *g* for 3 min (MiniSpin, Eppendorf AG, Hamburg, Germany). The supernatants were mixed 1:10 (*v*/*v*) with 1× treatment buffer, from which 3 μL was added into the gel pocket of the Bio-Rad 4–20% Criterion™ TGX Stain-Free™ Precast Gels. The Broad Range™ Unstained Protein Standard was used as the molecular weight marker. The running time was 30 min, followed by staining using Coomassie Brilliant Blue R-250. Finally, gel images were obtained using an EZ Imager (Gel Doc™ EZ Imager—Bio-Rad). Protein bands and their intensities were calculated using Image Lab Software. SDS-PAGE was performed in duplicate, with each sample being prepared two times independently.

### 2.6. Color

The colors of the protein isolates were measured using the Digi Eye system (VeriVide Limited, Leicester, UK) and a Nikon D90 camera (Nikon Metrology GmbH, Düsseldorf, Germany). The International Commission on Illumination (CIE) L*a*b* method was used to measure the parameters lightness (L*), green-red (a*), and blue-yellow (b*). The white color from the calibration board was used as the white reference for comparison among samples. The total color difference ($\Delta E_{ab}^{*}$) compared to the white reference board was calculated according to the CIE76 formula (Equation (1)). The color determination was performed in triplicate.

(1) $$\Delta E_{ab}^{*}=\;\sqrt{{\left(L_{2}^{*}-L_{1}^{*}\right)}^{2}+{\left(a_{2}^{*}-a_{1}^{*}\right)}^{2}+{\left(b_{2}^{*}-b_{1}^{*}\right)}^{2}}$$

### 2.7. Particle Size

The particle size distribution of all pea protein isolates was determined using a MasterSizer S Long Bed Version 2.19 equipped with a QS Small Volume Sample Dispersing Unit DIF2021 (Malvern Panalytical Ltd., Malvern, UK). The sample was dispersed in 1-Butanol for 2 min at 3000 rpm before measurements. After another minute, a second measurement was conducted. The measuring range was set at 300 RF 0.05–900 µm. The particle size was based on Mie theory with a refractive index of 1.33, using an index of 0.1 for dispersion media and 1.56 for the dispersed phase, with an imaginary proportion of 0.1.

### 2.8. Functional Properties

All analyses of functional properties were performed in duplicate.

#### 2.8.1. Protein Solubility

The protein solubility measurements at pH 4.5 and 7.0, respectively, were performed according to Morr and German [31]. The soluble protein content was determined photometrically at 550 nm following the Biuret method [32] using bovine serum albumin (BSA) as the standard for calibration. 

#### 2.8.2. Foaming Capacity

The foaming capacity was analyzed at pH 4.5 and 7.0 according to Phillips and Haque [33] using a whipping machine (Hobart N50, Hobart GmbH, Offenburg, Germany). Briefly, 5% (*w*/*v*) dispersions were whipped (580 rpm) for 8 min and the foaming capacities were determined as the relation between the initial and final volume.

#### 2.8.3. Emulsifying Capacity

The emulsifying capacity was determined according to Wang and Johnson [34] and García Arteaga, et al. [35] at pH 4.5 and 7.0. Briefly, 10 mL min^−1^ oil was added to a dispersion (1% *w*/*w*) in a 1 L reactor equipped with an Ultra-Turrax instrument and a conductivity meter. The volume of added oil was used to calculate the emulsifying capacity (mL oil/g sample). 

### 2.9. Sensory Analysis

#### 2.9.1. Sample Preparation

A 2% sample solution (1.7% protein, *w*/*w*) was prepared with tap water for each PPI. The respective samples were adjusted to pH 7.0 with 1 mol/L NaOH and coded using three-digit random numbers. 

#### 2.9.2. Sample Evaluation

The sensory evaluation was conducted according to DIN 10967-1-1999 and as described by García Arteaga, et al. [35]. Briefly, a trained panel evaluated attributes regarding retronasal aromas and tastes of the different PPIs. From each sample solution, 20 mL was presented at room temperature in a glass cup and in random order. The sensory evaluation was split into two evaluation sessions. In the evaluation sessions, each panelist evaluated six and seven samples, respectively. The panelists assessed the samples according to the following attributes: *fatty* (2-nonenal); *green* (hexanal); *earthy* (geosmin); *roasty* (2-acetylpyrazine); *pea-like* (2-isopropyl-3-methoxypyrazine); *metallic* ((trans)-4,5-Epoxy-(E)-decenal); *malty*; *nutty* (2,5-dimethylpyrazine). Additionally, panelists assessed the samples according to tastes such as *bitter*, *sweet*, *salty*, *astringent*, *mouth-coating*, and overall intensity. The intensities were scored from 0 (not perceivable) to 10 (very intense). 

### 2.10. Principal Component Analysis

A principal component analysis (PCA) is a multivariate statistical data analysis tool used to simplify the variability of data with a reduced number of dependent variables. A PCA (correlation matrix) was used to evaluate the similarities among isolates regarding their protein content, fat content, color, particle size, and functional properties. A covariance PCA was used to evaluate the aroma and taste. The PCA plots were performed using the software OriginPro 2018b. 

### 2.11. Statistical Analysis

Protein extractions were performed once and the resulting isolates were used for further analyses. Due to the low protein yields, all analyses were performed in duplicate, unless stated otherwise, and the results are expressed as mean values ± standard deviations. Non-parametric statistical analyses were performed due to the low number of replicates. The Kruskal–Wallis test was used to determine statistical differences among the cultivars. Dunn’s test with Bonferroni correction for *p*-values was used as a test for multiple comparisons. The results of the sensory analysis were analyzed using one-way ANOVA followed by Tukey’s post hoc test. A Kendall correlation coefficient was used to determine correlations between physicochemical, functional, and sensory properties. All statistical analyses were performed using OriginPro 2018b and were considered statistically significant at *p* < 0.05. The raw data are available as Mendeley Data [36].

## 3. Results and Discussion

### 3.1. Chemical Composition and Protein Yield

Table 2 shows the chemical compositions of the flours and PPIs, as well as the protein yields after spray drying. 

#### 3.1.1. Pea Flours

The protein contents of the dehulled pea flours ranged from 21.3% to 27.2%, similar to values obtained by Barac and Cabrilo [18] and Nikolopoulou and Grigorakis [6]. The flour from RLPY cultivar showed the highest protein content (27.2%), whereas the flour from Greenwich had the lowest protein content at 21.3%. The ash and fat contents ranged from 2.5% to 3.6% and from 1.9% to 2.5%, respectively. The flour from the Florida cultivar showed the highest fat content of 2.7%, whereas the flours of Dolores, Ostinato, Kalifa, RLPY, and Orchestra cultivars showed the lowest amounts at 1.9%. The flour from Navarro had the highest starch content, while RLPY had the lowest. The protein and starch contents obtained in this study were within the ranges of different cultivars investigated in other studies [22,37].

#### 3.1.2. Pea Protein Isolates

The protein contents of the pea protein isolates (PPIs) ranged from 83.5% to 90.3%. The PPI obtained from the RLPY cultivar showed the highest protein content, while the one from Navarro showed the lowest. The protein contents were in the same range as in other studies [17,18]; however, other studies obtained higher protein yields (62–89%), probably attributed to the drying technique, as high losses are common during spray drying [38]. It is worth mentioning that protein isolation at industrial scale might result in higher yields when the drying kinetics are correctly determined [39,40]. The highest protein yield was 62.2 g protein kg^−1^ seed^−1^ obtained from the Orchestra cultivar, followed by Florida with 59.2 g kg^−1^. The lowest protein yields were obtained from Navarro and Greenwich cultivars at 33.8 g kg^−1^ and 34.8 g kg^−1^, respectively. The ash contents of the PPIs varied from 5.3% to 8.5%, probably due to formation of salts (NaCl) after adjusting the pH during the different process steps. The fat contents ranged from 4.7% to 9.0%, with the Greenwich isolate having the highest fat content and Dolores isolate the lowest. The PPIs without a de-fatting step had higher lipid contents, probably due to the protein–lipid interactions during the extraction; Gao and Shen [41] showed that PPIs extracted after AE-IEP had predominantly hydrophobic β-sheets in their protein structures that could promote these interactions [42]. Furthermore, the increase in fat content might promote lipid–protein interactions in the isolates, which may lead to a higher hydrophobic character of the complexes, resulting in lower protein solubility. Their interaction may also reduce the availability of lipophilic groups, limiting the absorption of fat [43]. The color, aroma, and functionality might be also affected by the fat content, especially after lipid oxidation by lipoxygenase [44].

### 3.2. Molecular Weight Distribution

**Figure 1:**
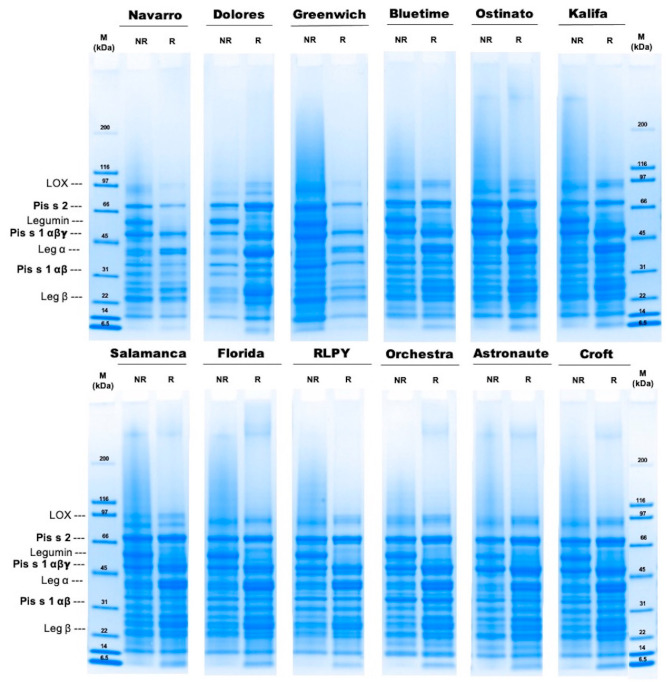
Molecular weight distribution of pea protein isolates from different cultivars, as determined by SDS-PAGE under non-reducing (NR) and reducing (R) conditions. Pis s2, Pis s1 αβγ, and Pis s2 αβ correspond to the allergen fractions from convicilin, mature vicilin- αβγ, and vicilin-αβ, respectively. M: molecular weight standard indicated in kilodalton (kDa).

Gel electrophoresis was performed under non-reducing and reducing conditions to reveal differences within the protein composition of the isolates from the different cultivars (Figure 1). The protein fractions ranged from 93 to 6.5 kDa. Three major fractions were identified in both conditions. Under non-reducing conditions, fractions around ~65 kDa, ~53 kDa, and ~45 kDa were most prominent, while under reducing conditions, 53 kDa proteins were absent and the intensity of the ~39 kDa fraction increased. Bands around 86 and 91 kDa may have been due to convicilin precursors and lipoxygenase (LOX), respectively [16,45]. The most visible difference between non-reducing and reducing conditions was observed for all protein isolates around the 50–56 kDa region, which might correspond to legumin [16,18]. Legumin consists of two polypeptides, one acid (Leg α) and one basic (Leg β) subunit, connected via disulfide bonding. These subunits were found at around 37–40 kDa for Leg α and 19–22 kDa for Leg β, with higher intensities under reducing conditions. 

Among the different protein fractions, the allergens Pis s2 and Pis s1 were investigated in detail. Table 3 shows the protein band intensities for each of the allergen fractions. These allergens lack cysteine residues, hindering the formation of disulfide bonds [14]. For this reason, the allergen protein fractions were expected to appear under both conditions. 

Convicilin Pis s2. The average molecular weight of the Pis s2 fraction was around 65 kDa, and values were not significantly different among the isolates. The isolate from Orchestra showed the strongest intensities under both conditions. In contrast, the Kalifa isolate and the Navarro isolate showed the lowest intensities under non-reducing and reducing conditions, respectively. The intensity of this protein fraction increased slightly under reducing conditions for all isolates, except for the Navarro and Greenwich isolates, where the intensity of the bands was slightly lower.

Vicilin Pis s1. The mature allergen fraction (vicilin-αβγ) was around 45 kDa under both conditions and was not significantly different among the isolates. Under non-reducing conditions, the Navarro isolate showed the strongest intensity. On the other hand, the RLPY and Florida isolates showed the lowest intensities. Under reducing conditions, vicilin-αβγ from Astronaute isolate showed the highest intensity, whereas the one from the Greenwich isolate showed the lowest. Vicilin-αβγ can go through post-translational cleavage, resulting in different fractions [46]. From these proteolytic fragments, vicilin-αβ (~32 kDa) was shown to bear a high allergenic potential [23]. Besides the Orchestra isolate, all isolates showed lower intensities of the vicilin-αβ fractions compared to the mature fraction and were not significantly different. 

The intensities of the protein and allergen fractions can differ among legume cultivars, while the intensity of the allergen fractions specifically can give an indication of the allergenic potential [14,18,47]. Overall, the Orchestra isolate showed slightly stronger intensities for the potential allergen fractions compared to the other isolates, whereas the isolate from Navarro had the lowest intensities for these fractions. However, the allergen fraction intensities were not significantly different among isolates. It is known that the globulin-to-albumin and legumin-to-vicilin ratios change throughout seed development [48], which could affect the presence and intensity of potential allergens. Even under the same environment, harvesting, and storage conditions, the variation among proteins in pea cultivars can be very large [14].

### 3.3. Color

Table 4 shows the color values of the samples and the white reference. The lightness (L*) levels among isolates were significantly different; the isolate of Orchestra cultivar showed significantly higher lightness (90.6) than the Bluetime (86.8) isolate. The Greenwich, Bluetime, and Croft isolates showed the lowest a* values, which corresponded to their cotyledon green color; however, only the isolate from Croft was significantly different to the isolates from Salamanca and Astronaute cultivars. In contrast, the isolate from the Navarro cultivar showed higher b* values, suggesting a stronger yellow color. The total color difference (ΔE*_ab_) allows for quantification of the colors and allows comparison between samples; the lower the ΔE*_ab_ value, the whiter the isolate is. All ΔE*_ab_ values ranged between 19.2 and 23.4. According to the lowest difference from the white reference, the isolates from the Dolores and Greenwich cultivars were most white, while the isolate from Navarro cultivar was least white.

### 3.4. Particle Size

Spray drying is one of the most common methods for drying protein solutions on an industrial scale. However, protein structures are to known to be affected by spray drying due to applied temperatures, vaporization, and the air–water interface.

These effects can cause protein denaturation and further aggregation of the exposed hydrophobic regions, which can affect the particle size of the dried proteins [20]. The particle size, in turn, is known to affect the physicochemical properties of proteins [49,50]. The particle sizes of the PPIs, described as the average volume weighted mean (d_4,3_), are shown in Table 5. The average d_4,3_ of the cultivar isolates was 11.9 µm. Of all protein isolates, the Florida protein isolate showed the largest d_4,3_ at 18.8 µm, followed by the Dolores and Croft isolates. The isolate from RLPY showed the smallest d_4,3_ at 7.5 µm, followed by Ostinato and Astronaute isolates. The different particle sizes among the investigated cultivar isolates might lead to differences in physicochemical behavior as a result of different particle morphologies [51].

### 3.5. Functional Properties

High functional properties of PPIs are desired to increase their usage as ingredients in different plant-based food products. Table 5 shows the results of the functional properties.

#### 3.5.1. Protein Solubility 

At pH 4.5, the Navarro isolate showed the highest protein solubility at 10.3%, which was different to the isolate from Florida (0.9%), Orchestra (1.5%), and Croft (0.0%) cultivars. On the other hand, at pH 7.0, the Orchestra isolate showed the highest protein solubility (61.8%), followed by the Dolores (60.8%) and Ostinato (60.4%) isolates. Overall, the protein solubility levels at pH 7 were similar among the isolates. Other studies have shown similar solubilities or even values up to 80% at pH 7.0 [17,18,19]. The protein solubility level is related to extraction and drying methods; for example, isolates obtained after alkaline extraction and isoelectric precipitation have lower solubility than those obtained after salt-induced extraction [17]. Moreover, in contrast to lyophilization used in previous studies, spray drying leads to higher protein denaturation, increasing hydrophobic protein–protein interactions, and thus reducing overall protein solubility [52]. High protein solubility levels are, however, essential for beverage and dairy-alternative applications; treatments such as proteolysis or the addition of _L_-Arginine and sodium carbonate are known to improve protein solubilities of PPIs [22,35].

#### 3.5.2. Emulsifying Capacity

The isolate from Navarro showed the highest emulsifying capacity at pH 4.5 with 405 mL g^−1^, while the one from Dolores and Florida showed the lowest. There were significant moderate correlations between the emulsifying capacity at pH 4.5 and both the protein solubility at pH 4.5 (r = 0.50) and the protein content (r = −63). On the other hand, the emulsifying capacity at pH 7.0 showed a significant positive moderate correlation with the protein content (r = 0.45). Thus, at neutral pH, the RLPY isolate showed the highest emulsifying capacity at 835 mL g^−1^ and was highly different from the isolates from Navarro and Astronaute cultivars. Hydrophobic residues are essential to facilitate protein oil interactions [53], however a high number of protein–protein interactions would form aggregates hiding hydrophobic residues, thus hindering the ability to interact with oil. These aggregates might be formed during spray drying, thus increasing particle size. However, no significant correlations were found between the emulsifying capacity and the particle size. Moreover, the vicilin/legumin ratio plays an important role in the formation of emulsions; Barac and Cabrilo [18] showed that the lower the ratio is, the higher the emulsifying capacity of the isolate, especially at neutral pH ranges. Although electrophoretic results showed no significant differences among allergens or overall in the electrophoretic patterns, further quantification of the fractions might be necessary to determine correlations with the functional properties.

#### 3.5.3. Foaming Capacity

At pH 7.0 no foam formation was observed, whereas at pH 4.5 all isolates showed an average foaming capacity of 866%. These results are in contrast to the results of Chao and Aluko [54], who obtained higher foaming capacities the further the pH moved away from the isoelectric point. On the other hand, Gharsallaoui and Cases [55] suggested that close to the isoelectric point (pH 4.5), pea globulins are more surface-active and a reduction in the electrostatic charge of the protein molecules might result in electrostatic repulsion, in turn increasing adsorption. The latter is important for the formation of foam [56] and might explain the foaming capacity at pH 4.5 for the cultivars investigated in this study. Another explanation is that the fat content in the PPIs might have acted as an antifoam agent. In order to destroy a foam film, the hydrophobic particle droplets that emerge from the aqueous phase into the air–water interface are critical [57]. At pH 7, the hydrophobic protein surfaces facilitate the entrance of the fat droplets, leading to defoaming. At pH 4.5, the hydrophobic side chains of the proteins are hidden, hindering the penetration of the fat droplets in the foam films. 

**Figure 2:**
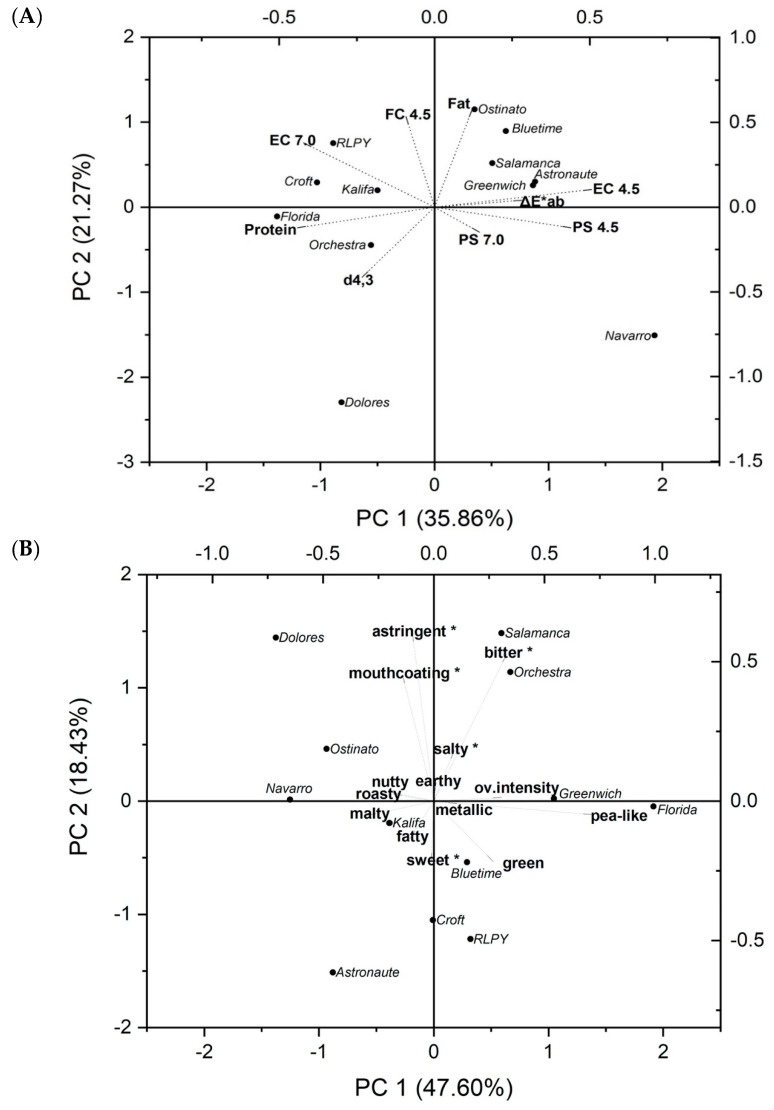
Biplot of (**A**) physicochemical properties and (**B**) sensory profiles of pea protein isolates from different pea cultivars. Attributes with an asterisk (*) refer to taste. PS: protein solubility; EC: emulsifying capacity; FC: foaming capacity; ΔE*_ab_: color difference compared to a white reference; d_4,3_: particle size. The numbers represent the pH (7.0 or 4.5) in which the analysis was performed.

A principal component analysis (PCA) was applied to analyze the relationships among the different cultivars and their colors, protein and fat contents, particle sizes, and physicochemical properties. Figure 2A shows a biplot of principal component (PC)1 and PC2 using the standardized scores for the isolates. The first two components of the PCA explained 57.13% of the total variance. The protein content (−0.44) and emulsifying capacity at pH 4.5 (0.51) had the strongest influence on PC1. On the other hand, the fat content (0.58) and foaming capacity (0.54) had the strongest influence on PC2; moreover, on the negative quadrant of the PC2, the particle size showed a strong influence (−0.41). 

The isolate from Navarro cultivar scored the highest for PC1 (1.93), opposite to the isolates from Kalifa, RLPY, and Croft. This is in agreement with the emulsifying capacities shown in Section 3.5. Furthermore, the Dolores isolate scored the highest in the PC2 (−2.30), followed by Navarro (−1.51), as they showed lower fat contents among the isolates. Negative moderate correlations were found between the protein content and the protein solubility (pH 4.5) and emulsifying capacity (pH 4.5). On the other hand, the protein content was significantly positive and moderately correlated with the emulsifying capacity at pH 7.0. The particle size showed no significant correlations to other investigated attributes. When replacing a raw material in an existing product, not only are the composition and functionality important, but the color should be also considered, as it can affect the perception of the product by the consumer; for this reason, the ΔE*_ab_ of the isolates was included in the PCA. However, the ΔE*_ab_ showed low influence on any of the components. 

The PCA shows two clusters plus two outliers. The isolates from RLPY, Croft, Kalifa, Florida, and Orchestra cultivars formed the first cluster; on the opposite side, isolates from Ostinato, Bluetime, Salamanca, Astronaute, and Greenwich cultivars formed the second cluster. These clusters suggest that the physicochemical characteristics are probably more similar and one cultivar could be replaced with another from the same cluster. On the other hand, the isolates from Navarro and Dolores were found to be further away from all other isolates, which might hinder the replacement of these cultivars. Moreover, the isolates should be chosen by considering the requirements of the final products. For example, the RLPY isolate could be used in applications with neutral pH, such as dairy alternatives, as it is plotted as having higher protein content, high emulsifying capacity (pH 7.0), and moderate protein solubility (pH 7.0); however, its application at low pH values is inappropriate due to its lower protein functionality. On the other hand, the Navarro isolate might be better suited in applications with acidic pH values, such as in plant-based mayonnaise.

### 3.6. Sensory Analysis 

A principal component analysis was applied to analyze relationships between samples and retronasal aroma attributes and taste profiles (Figure 2B). PC1 and PC2 represented 66.03% of the total variance; the following values represent the coefficient values (influence) of the attributes and the scores of the isolates from each cultivar. 

Aroma. According to a one-way ANOVA, *pea-like* was the only aroma attribute with a significant difference among isolates and showed the strongest influence on PC1 (0.71), followed by *malty* (−0.30) and *green* (0.27) aromas. For PC2, the *green* aroma showed the strongest influence among all aroma attributes (−0.22). The *metallic*, *earthy*, *roasty*, and *nutty* attributes showed almost no influence on any of the components. The isolates from Dolores (−1.38) and Navarro (−1.25) scored the lowest for PC1, which suggests these isolates were perceived to have the least *pea-like* aroma. In contrast, Florida (1.92) and Greenwich (1.05) isolates scored the highest for this component, indicating a stronger *pea-like* aroma. The *pea-like* aroma is known to be well-perceived because of the low thresholds of 2-isopropyl-3-methoxypyrazine [58], which might explain its strong influence. The PCA showed the isolates from Greenwich, Florida, RLPY, and Croft cultivars as being closer to the *green* attribute, in agreement with the results from the sensory analysis. The *green* aroma originating from hexanal is a characteristic oxidation product of fatty acids, in particular of linoleic and linolenic acids catalyzed by LOX [44]. Higher activity of this enzyme could increase the *green* aroma perception; however, there was only a moderate negative correlation (r = −0.54) between the *green* aroma and the LOX band intensities under reduction conditions. Although it has also been mentioned that green cultivars have higher levels of hexanal [8], there was no significant correlation between the a* color and *green* aroma.

Environmental and genetic conditions might affect the production and degradation rates of the aroma compounds, which would result in higher or lower aroma perceptions [8,59]. Using HC/MS analysis, Azarnia and Boye [8] found that the concentrations of volatile compounds depended on the cultivar, crop year, storage, and processing conditions. Specific processing methods such as enzymatic treatment or fermentation might be useful to reduce some of the characteristic off-flavors of pea isolates. However, other aroma compounds might be generated or enhanced and might further increase or decrease consumer acceptance [4,60,61]. Furthermore, methoxypyrazines are very stable during fermentation due to their chemical nature, and therefore are very difficult to remove or reduce [62]. Therefore, pea cultivars low in *pea-like* aroma, such as from Dolores or Navarro, are recommended to be used for production of PPIs with sensory appeal. 

Taste. The *bitter* attribute was the only significant taste attribute according to one-way ANOVA. The *bitter* (0.53) and *astringent* (0.60) tastes had the strongest influence on PC2. As shown in the PCA, the PPIs from Salamanca and Orchestra scored highest for bitter taste. In contrast, the PPI from Astronaute scored lowest for *bitter* taste and was significantly less *bitter* than the PPI from Salamanca. The Dolores isolate scored the highest for *astringent* taste, together with Salamanca and Orchestra isolates. Moreover, *salty* and *sweet* tastes had little influence on either component, which suggests that the intensity of these tastes was lower and similar among the samples. A high *bitter* taste for a PPI might hinder its application in food products; thus, several methods have been investigated to reduce the bitterness of legume protein isolates [61,63].

Overall Intensity. The isolate from Florida cultivar showed the highest overall intensity, whereas the isolate from Dolores showed the lowest intensity. However, the overall intensity levels among the PPIs were not significantly different. The overall intensity was moderately correlated with the *pea-like* (r = 0.66), *green* (r = 0.43), and *malty* (r = −0.47) aroma, which suggests that these compounds were characteristic of the isolates, as mentioned previously in the aroma section.

## 4. Conclusions

Peas are a valuable source of protein and are increasingly used in plant-based products; however, due to the large number of different cultivars, most of them have not been characterized regarding their chemical composition, functional properties, and sensory profiles. In this study, all these aspects were investigated for 12 cultivars grown in Germany and France.

Our study shows that the chemical composition of flour and isolates from the cultivars are slightly different. The main allergen fractions were present in all the PPI and showed no significant differences. The PCA showed two cluster of cultivars regarding the physicochemical and functional characteristics; however, these clusters were not found in the sensory profile PCA. This suggests that although some isolates could be substituted interchangeably for the same products with regard to their similar functionalities, the flavor of these products could be affected. However, only the *pea-like* and *bitter* aromas were significantly different among isolates. The cultivars Salamanca and Astronaute are the most used cultivars in Germany; they showed similarities according to the physicochemical-functional PCA cluster; however, Salamanca isolates had a significant higher bitter taste and slightly higher *pea-like* aroma than Astronaute. These differences should be considered for targeted product developments as they might influence the acceptance by consumers. The usage of cultivars such as Navarro and Dolores should be carefully considered, as their isolates are mostly different to the other cultivars investigated in the present study.

The obtained PPI might be used in the food industry, especially under neutral conditions (pH 7.0), except when foaming is required; however, when designing a food product in the acidic range at pH 4.5, the specific selection of a suitable cultivar might be more important. Differences with laboratory and commercial processing of PPI should be considered; although spray drying was used in this study, larger spray-dryers may affect the physicochemical, functional and sensory properties of the isolates. The results of this study highlighted the importance of a tailored selection of cultivars for protein extraction as well as the suitability of pea cultivars for specific food applications.

## Figures and Tables

**Table 1:** List of pea cultivars investigated in this study.

Cultivar	Harvest Year	Place of Cultivation	Cotyledon Color	Admitted in
Navarro	2018	Malchow/Mecklenburg-Vorpommern	yellow	Germany
Dolores	2015	Oderaue/Mecklenburg-Vorpommern	yellow	Germany
Greenwich	2018	Hohenlieth/Schleswig-Holstein	green	Great Britain
Bluetime	2018	Hohenlieth/Schleswig-Holstein	green	Great Britain
Ostinato	2018	Rodez/France	yellow	France
Kalifa	2017	Hohenlieth/Schleswig-Holstein	yellow	Breeding line
Salamanca	2018	Malchow/Mecklenburg-Vorpommern	yellow	Germany, Czech Republic, etc.
Florida	2015	Dreveskirchen/Mecklenburg-Vorpommern	yellow	Germany
RLPY141091	2018	Rodez/France	yellow	Germany
Orchestra	2018	Rodez/France	yellow	France, Germany
Astronaute	2018	Groß Kiesow/Mecklenburg-Vorpommern	yellow	France, Germany, etc.
Croft	2018	Hohenlieth/Schleswig-Holstein	green	Great Britain

**Table 2:** Chemical composition and protein yield of dehulled flour and protein isolates produced from different pea cultivars.

Cultivar	Dry Matter	Protein *	Ash 550 *	Fat *	Starch *	Protein Yield **
	[%]	[%]	[%]	[%]	[%]	[g kg^−1^]
*Dehulled Flour*						
Navarro	89.6 ± 0.0	22.1 ± 0.1	2.9 ± 0.0	2.3 ± 0.0	52.6 ± 0.2	-
Dolores	91.4 ± 0.0	26.5 ± 0.2	3.0 ± 0.0	1.9 ± 0.0	44.3 ± 2.5	-
Greenwich	90.9 ± 0.0	21.3 ± 0.2	2.7 ± 0.0	2.5 ± 0.0	48.2 ± 0.3	-
Bluetime	91.5 ± 0.1	22.4 ± 0.4	2.9 ± 0.0	2.2 ± 0.0	40.3 ± 0.5	-
Ostinato	91.2 ± 0.0	25.0 ± 0.2	3.6 ± 0.1	1.9 ± 0.0	47.6 ± 0.5	-
Kalifa	91.4 ± 0.1	24.2 ± 0.1	3.0 ± 0.0	1.9 ± 0.0	46.6 ± 0.1	-
Salamanca	90.8 ± 0.0	22.4 ± 0.1	2.8 ± 0.0	2.0 ± 0.0	49.2 ± 3.3	-
Florida	91.2 ± 0.1	24.8 ± 0.1	2.9 ± 0.0	2.7 ± 0.2	45.0 ± 4.6	-
RLPY 141091	91.3 ± 0.1	27.2 ± 0.0	2.8 ± 0.0	1.9 ± 0.0	32.5 ± 0.8	-
Orchestra	92.2 ± 0.1	26.3 ± 0.2	3.5 ± 0.2	1.9 ± 0.0	35.8 ± 0.3	-
Astronaute	91.2 ± 0.0	22.0 ± 0.0	2.5 ± 0.1	2.0 ± 0.1	45.3 ± 1.1	-
Croft	91.8 ± 0.1	22.5 ± 0.1	2.6 ± 0.0	2.1 ± 0.0	48.0 ± 2.2	-
*Protein Isolate*						
Navarro	93.0 ± 0.0	83.5 ± 0.4	5.3 ± 0.3	5.9 ± 0.0	-	33.8
Dolores	93.5 ± 0.1	89.5 ± 0.2	5.4 ± 0.1	4.7 ± 0.1	-	54.4
Greenwich	93.8 ± 0.0	83.6 ± 0.4	6.0 ± 0.6	9.0 ± 0.2	-	34.8
Bluetime	94.4 ± 0.0	84.1 ± 0.0	6.4 ± 0.4	8.4 ± 0.3	-	42.2
Ostinato	94.1 ± 0.0	86.0 ± 0.5	7.6 ± 0.4	7.1 ± 0.4	-	38.6
Kalifa	93.0 ± 0.0	86.9 ± 0.9	5.9 ± 0.1	7.0 ± 0.5	-	46.2
Salamanca	93.7 ± 0.6	85.0 ± 0.3	6.1 ± 1.0	8.7 ± 0.6	-	42.2
Florida	92.5 ± 0.0	87.4 ± 1.1	5.6 ± 0.1	7.4 ± 0.7	-	59.2
RLPY 141091	93.4 ± 0.0	90.3 ± 0.0	8.5 ± 0.7	7.3 ± 0.8	-	53.6
Orchestra	92.8 ± 0.3	87.1 ± 0.1	6.7 ± 1.1	6.2 ± 0.9	-	62.2
Astronaute	96.0 ± 0.2	86.4 ± 0.1	5.4 ± 0.1	7.8 ± 0.1	-	42.1
Croft	92.5 ± 0.1	86.7 ± 0.6	6.2 ± 0.1	7.8 ± 0.1	-	47.3

Results are expressed as means ± standard deviations (*n* = 2). No significant differences were found among cultivars within the same column (Dunn’s test with Bonferroni correction, *p* < 0.05). Note: * based on dry matter; ** based on protein content (g of protein/kg of seeds).

**Table 3:** Protein band intensities (Int) of globular protein allergens of pea protein isolates, namely convicilin (Pis s2), vicilin αβγ (Pis s1), and vicilin-αβ (Pis s1) from different cultivars as determined by sodium dodecyl sulfate–polyacrylamide gel electrophoresis.

Cultivar	Protein Band Intensity [Int]					
	Convicilin Pis s2		Vicilin αβγ Pis s1		Vicilin αβ Pis s1	
	NR	R	NR	R	NR	R
Navarro	185 ± 16	146 ± 79	308 ± 34	280 ± 73	81 ± 13	95 ± 50
Dolores	214 ± 5	367 ± 35	166 ± 22	282 ± 43	118 ± 5	97 ± 11
Greenwich	219 ± 47	185 ± 57	229 ± 55	256 ± 36	126 ± 16	113 ± 2
Bluetime	241 ± 17	263 ± 49	236 ± 66	311 ± 39	111 ± 7	127 ± 9
Ostinato	253 ± 31	343 ± 32	252 ± 66	361 ± 11	105 ± 19	116 ± 37
Kalifa	141 ± 6	205 ± 36	285 ± 47	392 ± 46	122 ± 8	153 ± 7
Salamanca	280 ± 2	363 ± 5	233 ± 51	350 ± 20	89 ± 3	88 ± 16
Florida	218 ± 16	283 ± 51	196 ± 88	330 ± 60	94 ± 1	72 ± 6
RLPY 141091	294 ± 13	379 ± 63	149 ± 25	285 ± 78	117 ± 8	106 ± 0
Orchestra	302 ± 50	421 ± 32	212 ± 78	398 ± 19	228 ± 38	180 ± 5
Astronaute	251 ± 34	365 ± 44	293 ± 72	411 ± 20	118 ± 28	99 ± 8
Croft	261 ± 55	372 ± 79	235 ± 79	353 ± 29	93 ± 7	79 ± 20

Results are expressed as means ± standard deviations (*n* = 2). No significant differences were found among cultivars within the same column (Dunn’s test with Bonferroni correction, *p* < 0.05). NR: non reducing conditions; R: reducing conditions.

**Table 4:** CIE lab color results from pea protein isolates from different cultivars and a commercial pea protein isolate.

Cultivar	Pea Isolate CIE Color			
	L*	a*	b*	ΔE*_ab_
Navarro	89.3 ± 0.4 _ab_	2.8 ± 0.1 _ab_	23.7 ± 0.2 _a_	23.4 ± 0.2 _a_
Dolores	88.5 ± 0.1 _ab_	1.9 ± 0.0 _ab_	19.1 ± 0.2 _a_	19.2 ± 0.1 _a_
Greenwich	88.2 ± 0.1 _ab_	0.6 ± 0.1 _ab_	19.2 ± 0.4 _a_	19.3 ± 0.3 _a_
Bluetime	86.8 ± 0.3 _a_	0.9 ± 0.1 _ab_	20.5 ± 0.7 _a_	21.0 ± 0.7 _a_
Ostinato	89.4 ± 0.3 _ab_	3.2 ± 0.2 _ab_	20.5 ± 0.4 _a_	20.4 ± 0.5 _a_
Kalifa	89.5 ± 0.3 _ab_	2.8 ± 0.0 _ab_	20.5 ± 0.2 _a_	20.3 ± 0.2 _a_
Salamanca	88.3 ± 0.2 _ab_	3.3 ± 0.1 _ab_	21.3 ± 0.3 _a_	21.5 ± 0.3 _a_
Florida	88.6 ± 0.3 _ab_	2.7 ± 0.2 _ab_	20.8 ± 0.2 _a_	20.8 ± 0.3 _a_
RLPY 141091	90.1 ± 0.2 _ab_	3.1 ± 0.1 _ab_	22.0 ± 0.2 _a_	22.0 ± 0.3 _a_
Orchestra	90.6 ± 0.6 _b_	2.6 ± 0.3 _ab_	20.9 ± 0.4 _a_	20.3 ± 0.5 _a_
Astronaute	88.2 ± 0.3 _ab_	3.5 ± 0.1 _a_	22.8 ± 0.3 _a_	22.9 ± 0.3 _a_
Croft	87.3 ± 0.3 _ab_	-0.5 ± 0.0 _b_	19.9 ± 0.2 _a_	20.3 ± 0.3 _a_

Results are expressed as means ± standard deviations (*n* = 3). Subscripts with different letters indicate significant differences within the same column (Dunn’s test with Bonferroni correction, *p* < 0.05). Note: ΔE*_ab_: color difference compared to a white reference.

**Table 5:** Physicochemical and functional properties of pea protein isolates from different pea cultivars.

	Particle Size	Protein Solubility **		Emulsifying Capacity		Foaming Capacity
Cultivar	d_4,3_	pH 4.5	pH7.0	pH 4.5	pH 7.0	pH 4.5
	[μm]	[%]	[%]	[mL g^−1^]	[mL g^−1^]	[%]
Navarro	13.19 ± 0.56	10.3 ± 0.2	51.5 ± 0.9	405 ± 1	600 ± 7	805 ± 0
Dolores	15.81 ± 0.06	7.4 ± 0.0	60.8 ± 2.8	340 ± 7	706 ± 14	808 ± 4
Greenwich	12.82 ± 0.19	8.8 ± 1.3	55.4 ± 3.4	396 ± 2	734 ± 7	839 ± 36
Bluetime	9.20 ± 0.49	7.7 ± 0.2	53.8 ± 2.4	365 ± 1	710 ± 8	915 ± 0
Ostinato	7.86 ± 0.02	8.3 ± 1.9	60.4 ± 1.9	385 ± 14	787 ± 32	959 ± 10
Kalifa	13.55 ± 1.53	7.3 ± 0.0	40.0 ± 2.1	354 ± 1	747 ± 3	911 ± 40
Salamanca	10.15 ± 0.40	5.9 ± 0.6	48.6 ± 3.6	378 ± 11	744 ± 2	835 ± 0
Florida	18.84 ± 1.31	0.9 ± 1.3	41.3 ± 7.1	340 ± 7	781 ± 23	884 ± 14
RLPY 141091	7.53 ± 0.01	2.3 ± 0.6	52.6 ± 2.8	359 ± 5	835 ± 7	874 ± 13
Orchestra	11.31 ± 0.21	1.5 ± 0.0	61.8 ± 6.0	366 ± 1	790 ± 6	835 ± 9
Astronaute	7.94 ± 0.29	6.3 ± 0.3	52.4 ± 0.9	381 ± 7	681 ± 23	858 ± 23
Croft	14.66 ± 1.35	0.0 ± 0.0	43.6 ± 5.1	355 ± 0	790 ± 24	861 ± 6

Results are expressed as means ± standard deviations. No significant differences were found among cultivars within the same column (Dunn’s test with Bonferroni correction, *p* < 0.05). The particle size was based on Mie’s theory (RI1.33). Note: d_4,3_: volume weighted mean; ** based on protein content.

## Data Availability

Raw data are available as Mendeley Data doi:10.17632/ywpjs6h3jr.1.
